# Real-world data of fracture rates and musculoskeletal disorders for patients living with osteogenesis imperfecta

**DOI:** 10.1093/jbmrpl/ziaf124

**Published:** 2025-07-21

**Authors:** Erru Christy Yang, Osman Ciğeroğlu, Rupal N Gupta, Ying Shen, Soraya Sader, Heather M Byers, Mahim Jain, Jeanne M Franzone

**Affiliations:** Ultragenyx Pharmaceutical Inc., Global HEOR, Novato, CA 94949, United States; Ultragenyx Pharmaceutical Inc., Global HEOR, Novato, CA 94949, United States; Ultragenyx Pharmaceutical Inc., Global HEOR, Novato, CA 94949, United States; Tianjin Happy Life Technology Co., Ltd., Tianjin, 100083, China; Ultragenyx Pharmaceutical Inc., Global HEOR, Novato, CA 94949, United States; Ultragenyx Pharmaceutical Inc., Global HEOR, Novato, CA 94949, United States; Kennedy Krieger Institute, Johns Hopkins Medicine, Baltimore, MD 21205, United States; Nemours Children’s Hospital, Department of Orthopedic Surgery, Wilmington, DE 19803, United States; Kennedy Krieger Institute, Johns Hopkins Medicine, Baltimore, MD 21205, United States; Nemours Children’s Hospital, Department of Orthopedic Surgery, Wilmington, DE 19803, United States

**Keywords:** osteogenesis imperfecta, health economics, general population studies, fracture risk assessment, bone and mineral diseases-other

## Abstract

Osteogenesis imperfecta (OI) is a genetic disease characterized by bone fragility and recurrent fractures. Fracture rate has been advocated as an important endpoint for evaluating investigative therapies in OI, but real-world data on fracture burden in the OI population are lacking. This retrospective US claims database study aimed to quantify fracture rate and the occurrence of musculoskeletal disorders among patients with OI of all ages and in a subgroup of patients who were treated with ≥1 off-label therapies. 5722 patients with OI were identified in the IQVIA PharMetrics Plus database and 2095 (55.7% female; 35.1% peds; 20% with OI-related treatments; 87.1% commercial-insured) met eligibility criteria and were included in analyses. About 40% of patients with OI and 37% of those with off-label treatments had a fracture within 1 yr of diagnosis (index date for all OI) or treatment (index date for OI-treated). The annual average fracture rates of these groups were 0.84 and 0.98, respectively. Matched non-OI comparators had 3% fracture occurrence and a 0.03 annual fracture rate. Among patients with ≥1 fracture episode, the annualized median fracture rate was 2 per year for both OI and OI-treated cohorts and 1 for the non-OI cohort. Pediatric patients had the highest fracture occurrence and fracture rate, both among patients with OI and the subgroup of treated OI patients. Musculoskeletal disorders and diagnosed pain were more frequent in OI patients compared with the non-OI cohort, especially among pediatric age groups and adults 18-<45 yr. Pain and musculoskeletal disorders occurred frequently among OI patients, regardless of fracture occurrence, demonstrating impacts of OI beyond those that can be attributed to fractures. Together, these results highlight a high burden of fracture and other musculoskeletal disorders in a commercially insured population of OI patients, which are not effectively managed by current management strategies or off-label treatments.

## Introduction

Osteogenesis imperfecta (OI) refers to a group of genetic conditions characterized by low bone mass and fragile, easily fractured bones.^1–4^ Osteogenesis imperfecta is a lifelong condition that significantly impacts quality of life and lifespan.^5^ Clinical presentation ranges from a mild phenotype of moderately increased fracture risk with normal to slightly shortened stature to a severe phenotype characterized by extreme fracture rates, subsequent skeletal deformities, and in some cases, perinatal lethality.^4^^,^^6^ Individuals with OI experience recurrent fractures throughout their lifetime, particularly in long bones, vertebrae, and ribs.^2^^,^^7^ While other phenotypes are also present, the greatly increased fracture rate is the most common, leading to multiple emergency room (ER) visits and significant disease burden due to pain, impaired mobility, and progressive skeletal deformation.^6^^,^^8–11^

Bone fragility in OI most commonly results from defects in the production, processing, or assembly of type I collagen fibers and/or the surrounding extracellular matrix.^6^^,^^12^ Patients with OI experience many musculoskeletal disorders beyond fractures, most of which are also related to defects in collagen and collagen extracellular matrix, including joint laxity, hypermobility, joint deterioration, knee pain, and muscle weakness.^13^ Patients may also experience progressive skeletal deformities, including bowing of the long bone segments of upper and lower extremities, a barrel-shaped chest, and spinal deformities, such as scoliosis, kyphosis, and basilar invagination.^6^ There are more than 20 types of OI, classified based on clinical and radiographic findings, inheritance pattern, and genetic mutations.^12^^,^^14^ The vast majority of individuals (~85%) with OI have pathogenic variants in the type I collagen coding genes.^1^ Regardless of the suspected type or genetic diagnosis, OI is diagnosed as a single disease in the US and billable with the ICD-10-CM diagnosis code of OI Q78.0.

There are currently no Food and Drug Administration-approved treatments for OI and management focuses on alleviating symptoms and early developmental intervention to increase BMD, reduce fracture risk, and improve quality of life.^6^^,^^15^ Pharmacological management relies on off-label use of therapeutic agents initially developed for conditions of altered bone metabolism, with the rationale that medications that increase bone density and reduce bone turnover might favorably influence clinical outcome and reduce fracture risk.^16^^,^^17^ These agents include bisphosphonates (IV or oral), denosumab (a fully human monoclonal antibody against RANKL), teriparatide (a parathyroid hormone analog), and romosozumab (a humanized anti-sclerostin monoclonal antibody).^18^^,^^19^ While multiple studies have shown an association between IV bisphosphonate treatment and decreased fracture rates in children, especially vertebral fractures,^15^^,^^20^^,^^21^ most studies of bisphosphonates in OI have been small and underpowered, and meta-analyses showed no significant reduction in fractures.^18^^,^^22^^,^^23^

Fracture rate has been advocated as an important endpoint for randomized-controlled trials of investigative therapies in OI, but a clear understanding of overall fracture risk and impact in OI patients is limited. While several clinical studies have assessed fracture rates among OI patients,^11^^,^^24^ there is a lack of recent data documenting fracture rate across all age groups of OI patients, in OI patients treated off-label with bisphosphonates or other agents, or in individuals unaffected by OI. Clinical literature also lacks a comprehensive assessment of musculoskeletal disorders of OI among different age groups. This US retrospective claims database study aims to quantify diagnosed fracture rate and the occurrence of musculoskeletal disorders among patients with OI in all age groups, and in a subgroup of patients who were treated with one or more off-label treatments. The results provide real-world data on fracture and musculoskeletal burden and unmet needs of patients with OI.

## Materials and methods

This retrospective study used the IQVIA Pharmetrics Plus database of adjudicated, deidentified, and integrated medical and pharmacy (retail and mail order) claims data for >150 million members from >70 US commercial health plans. The database uses the International Classification of Diseases, Tenth Revision, Clinical Modification (ICD-10CM) coding system to record patient medical information. Inclusion criteria for individuals with OI were at least 2 ICD-10-CM diagnosis codes for OI, OI Q78.0, at least 30 d apart (to ensure confirmation of disease). Exclusion criteria were any evidence of clinical trial participation and an OI diagnosis date that fell during the study period. A subgroup of patients with OI who had prescriptions for at least one of the off-label treatments of interest (bisphosphonates, denosumab, romosozumab, and teriparatide) were included in the OI treated cohort. A healthy comparator group was selected from individuals in the database with no OI diagnosis using 1:3 exact matching for age group, gender, payer type, and continuous enrollment (CE) start year.

Individuals in all groups were required to have ≥12 mo continuous medical enrollment between January 2016 and February 2020 (before COVID impacted period). Those in the OI treated cohort were required to have ≥12 mo continuous medical and pharmacy enrollment after the date of first prescription/injection. The index date for analyses was defined as the first day of the OI diagnosis for overall OI cohort and as the date of first prescription/injection for the OI treated cohort.

Fractures were assessed for 1 yr after the index date. Fracture occurrence and location were identified with ICD-10 diagnosis codes using an adapted fracture algorithm (Supplemental appendix). Diagnoses of fracture in the same body part within 90 d were grouped as the same episode. Results for each cohort were reported overall, by age group and by fracture status. The specific age groups were prespecified and differ between analyses, as indicated in the tables and figures.

## Results

### Patient selection and demographics

About 5722 individuals with ≥2 OI-related medical claims at least 30 d apart were identified. Of those, 2095 of whom met CE eligibility criteria and comprised the overall OI cohort (Figure S1). About 28% of these OI patients (*n* = 593) had prescriptions for treatments of interest during the study period, and 433 were eligible for inclusion in the OI treated cohort (Figure S1). The non-OI cohort includes 6285 individuals with no OI diagnosis during the study period. The cohorts were well matched by gender (~55% female), age (mean ~30 yr, ~35% pediatric), type of insurance (~87% to 91% commercial), and enrollment duration (mean 3.17-3.97 yr) (Table S1).

### Treatment patterns

About one quarter of the overall OI cohort (*n* = 510) had at least one treatment of interest during the CE period, most commonly bisphosphonates (21% overall and 87% of treated OI cohort). Annualized bisphosphonate administration frequency was around two for all OI patients and for those with or without fracture (Table 1). Individuals in the <18 yr age group were most likely to receive a treatment of interest (32%), followed by those over 25 yr (22%) and those 18-25 yr (13%) (Table 1).

**Table 1:** Proportion and annualized treatment frequency during CE by fracture status and age group.

**Treatment patterns by fracture status**			**Overall (*N* = 2095)**		**Without fracture (*N* = 1013)**		**With fracture (*N* = 1082)**	
			***N***	**%**	***N***	**%**	***N***	**%**
**Any treatment of interest**			510	24.3	156	15.4	354	32.7
**Bisphosphonates (BP)**			441	21.1	133	13.1	308	28.5
**Frequency (among pts with events; annualized)**			2.07	2.32	2.14	2.68	2.04	2.16
** Oral**			136	6.5	60	5.9	76	7.0
** Frequency (among pts with events; annualized; mean, SD)**			3.02	3.21	3.35	3.4	2.75	3.05
** IV**			313	14.5	75	7.4	238	22.0
** Frequency (among pts with events; annualized; mean, SD)**			1.61	1.63	1.12	1.19	1.76	1.72
**Other OI-related treatment**			91	4.3	31	3.1	60	5.6
** Denosumab**			68	3.3	27	2.7	41	3.8
** Romosozumab**			0	0.0	0	0.0	0	0.0
** Teriparatide**			32	1.5	5	0.5	27	2.5
**Treatment patterns by age group**	**Overall (*N* = 2095)**		**<18 yr** ***N* = 735**		**Age 18-25** ***N* = 241**		**Age >25** ***N* = 1119**	
	***N***	**%**	***N***	**%**	***N***	**%**	***N***	**%**
**Any treatment of interest**	510	24.3	234	31.8	32	13.3	244	21.8
**Bisphosphonates (BP)**	441	21.1	232	31.6	32	13.3	177	15.8
**Frequency (among pts with events; annualized; mean, SD)**	2.07	2.32	2.07	1.99	1.46	1.71	2.18	2.77
** Oral**	136	6.5	15	2.0	9	3.7	112	10.0
** Frequency (among pts with events; annualized; mean, SD)**	3.02	3.21	3.93	3.7	2.07	2.67	2.97	3.18
** IV**	313	14.9	218	29.7	24	10.0	71	6.3
** Frequency (among pts with events; annualized; mean, SD)**	1.61	1.63	1.94	1.76	1.17	1.14	0.76	0.8
**Other OI-related treatment**	91	4.3	2	0.3	1	0.4	88	7.9
** Denosumab**	68	3.3	2	0.3	1	0.4	65	5.8
** Romosozumab**	0	0.0	0	0.0	0	0.0	0	0.0
** Teriparatide**	32	1.5	0	0.0	0	0.0	32	2.9

### Fracture occurrence and annualized fracture rate

Fracture occurrence was low in the non-OI cohort and common among the OI cohorts; 40% of all OI patients and 45% of those with off-label treatments had a fracture within 1 yr of diagnosis (index date for overall OI) or treatment (index date for treated subgroup) (Table 2). The annual average fracture rates for these groups were 0.84 and 0.98, respectively. The non-OI cohort had 3% fracture occurrence and a 0.03 annual fracture rate (Figure S2). During the entire CE period (mean: 3.4 yr; median: 2.91 yr), 52% of patients with OI and 62% of those in the treated subgroup had a fracture, with annual average fracture rates of 0.58 and 0.71, respectively (Figure S2). Among patients with at least one fracture episode, the annualized median fracture rate in the first year was 2 for both OI cohorts and 1 for the non-OI cohort. During the entire CE period, annualized median fracture rates were 0.71, 0.73, and 0.3 for all OI, treated OI, and non-OI cohorts, respectively (Table 2).

**Table 2:** Fracture occurrence and number of fracture rate, first year post index, and entire continuous enrollment period.

	**During first year**			**During entire CE**		
	**All OI patients (*n* = 2095)**	**Treated OI patients (*n* = 433)**	**Non-OI cohort (*n* = 6285)**	**All OI patients (*n* = 2095)**	**Treated OI patients (*n* = 433)**	**Non-OI cohort (*n* = 6285)**
***N* with fracture rate**	842	194	164	1082	268	474
**% with fracture occurrence (%)**	40.2	44.8	2.6	51.6	61.9	7.5
**Average annual fracture rate among patients with ≥1 episode**	2.08	2.19	1.27	1.12	1.15	0.46
**Median annual fracture rate among patients with ≥1 episode**	2	2	1	0.71	0.73	0.3

Pediatric OI patients, especially those under 7 yr of age, had the highest fracture occurrence and rates regardless of treatment subgroup. Treated pediatric patients age <7 had higher fracture occurrence within the first year compared with overall OI patients in this age group (overall OI: 60% [*n* = 156], treated OI: 68% [*n* = 43]) (Table 3). Older pediatric patients (7-17 yr) with OI had the second highest fracture rates (overall OI: 54% [*n* = 258]; treated OI: 52% [*n* = 64]) (Tables 3 and 4). The age group trend of decreasing fracture rates with increased age was consistent through the adult age groups, as was the trend for higher fracture rates among the treated subgroups. An exception was the >65 yr age group, which had a decreased fracture frequency in the treated patients compared the overall OI cohort (20%, *n* = 5 vs 31%, *n* = 26) (Table 4). All cohorts had the highest fracture rates during the first year and similar treatment group trends for fracture rates throughout the CE period (Tables 3 and 4).

**Table 3:** Annualized fracture rate by age group among the overall OI cohort, first year post index, and entire continuous enrollment period.

	**During first year post index**					
**Age group**	**<7**	**7-17**	**18-25**	**26-44**	**45-64**	**>65**
**Overall Patients (*N*)**	260	475	241	511	523	85
** Mean**	1.39	1.20	0.74	0.55	0.60	0.58
** SD**	1.64	1.51	1.48	1.11	1.08	1.08
** Median**	1.00	1.00	0.00	0.00	0.00	0.00
** Min, Max**	0, 9	0, 8	0, 10	0, 7	0, 7	0, 6
**Patients with fracture occurrence (*N*, %)**	156 (60.0)	258 (54.3)	84 (34.9)	142 (27.8)	176 (33.7)	26 (30.6)
** Mean**	2.31	2.21	2.12	1.96	1.79	1.88
** SD**	1.53	1.41	1.83	1.30	1.16	1.18
** Median**	2.00	2.00	1.00	1.00	1.00	2.00
** Min, Max**	1, 9	1, 8	1, 10	1, 7	1, 7	1, 6
	**During entire CE period**					
**Age group**	**<7**	**7**-**17**	**18**-**25**	**26**-**44**	**45**-**64**	**>65**
**Overall Patients (*N*)**	260	475	241	511	523	85
** Mean**	1.04	0.82	0.50	0.37	0.40	0.50
** SD**	1.38	1.07	1.06	0.80	0.68	0.81
** Median**	0.59	0.44	0.00	0.00	0.00	0.00
** Min, Max**	0, 8.01	0, 6.08	0, 9.38	0, 7.36	0, 4.54	0, 3.17
**Patients with fracture occurrence (*N*, %)**	177 (68.1)	320 (67.4)	108 (44.8)	195 (38.2)	241 (46.1)	41 (48.2)
** Mean**	1.52	1.21	1.11	0.97	0.86	1.03
** SD**	1.43	1.10	1.35	1.04	0.77	0.90
** Median**	0.99	0.82	0.67	0.59	0.58	0.67
** Min, Max**	0.16, 8.01	0.14, 6.08	0.14, 9.38	0.14, 7.36	0.14, 4.54	0.14, 3.17

**Table 4:** Annualized fracture rate by age group among the OI-treated cohort, first year post index, and entire continuous enrollment period.

	**During first year post index**					
**Age group**	**<7**	**7**-**17**	**18**-**25**	**26**-**44**	**45**-**64**	**>65**
**Overall patients (*N*)**	63	124	28	47	146	25
** Mean**	2.05	1.16	0.75	1.06	0.51	0.32
** SD**	1.89	1.63	1.21	1.36	0.96	0.75
** Median**	2.00	1.00	0.00	1.00	0.00	0.00
** Min, Max**	0, 6	0, 7	0, 4	0, 6	0, 7	0, 3
**Patients with fracture occurrence (*N*, %)**	43 (68.3)	64 (51.6)	10 (35.7)	26 (55.3)	46 (31.5)	5 (20)
** Mean**	3.00	2.25	2.10	1.92	1.61	1.60
** SD**	1.54	1.64	1.10	1.29	1.06	0.89
** Median**	3.00	2.00	2.00	2.00	1.00	1.00
** Min, Max**	1, 6	1, 7	1, 4	1, 6	1, 7	1, 3
	**During entire CE period**					
**Age group**	**<7**	**7**-**17**	**18**-**25**	**26**-**44**	**45**-**64**	**>65**
**Overall patients (*N*)**	63	124	28	47	146	25
** Mean**	1.58	0.76	0.64	0.61	0.42	0.30
** SD**	1.60	0.96	1.05	0.87	0.69	0.52
** Median**	1.09	0.49	0.00	0.23	0.00	0.00
** Min, Max**	0, 7.16	0, 5.75	0, 4.44	0, 4.52	0, 3.36	0, 1.92
**Patients with fracture occurrence (*N*, %)**	56 (88.9)	91 (73.4)	13 (46.4)	30 (63.8)	69 (47.3)	9 (36)
** Mean**	1.78	1.04	1.37	0.96	0.89	0.83
** SD**	1.59	0.98	1.18	0.93	0.78	0.55
** Median**	1.33	0.70	0.97	0.71	0.67	0.75
** Min, Max**	0.2, 7.16	0.14, 5.75	0.29, 4.44	0.16, 4.52	0.14, 3.36	0.14, 1.92

**Figure 1:**
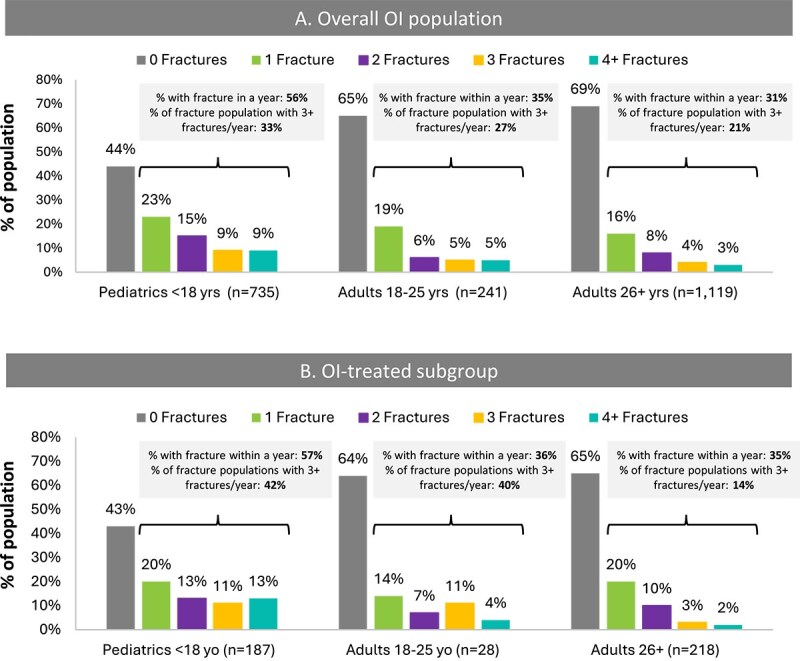
Proportion of patients by number of fractures within a year by age group. Graphs show the proportion of patients by number of fractures within a year (a measure of fracture burden), broken down by age group. (A and B) In the subgroup of treated patients. Overall, fracture burden was high in all age groups for treated and untreated OI patients and was highest for pediatric patients. The proportion of patients with 3 or more fractures was also high overall (ranging from 14% to 42%) and highest for pediatric patients (40%-42%).

More than 50% of pediatric patients and around 35% of all adult patients (≥18) experienced at least one fracture within a year and 37% and 16%, respectively, experienced at least two fractures (Figure 1). Among patients with OI reporting at least one fracture, 33% of pediatrics and >20% of adults had more than three fractures within a year (Figure 1A). Among patients reporting at least one fracture in the treated OI cohort, more than 42% of pediatrics and 40% of young adults (18-25 yr) had more than three fractures within a year (Figure 1B).

**Figure 2:**
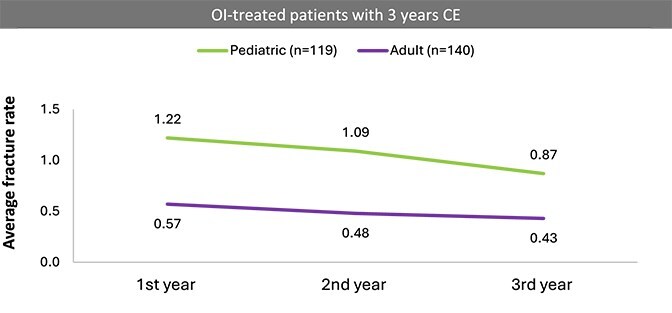
Annual fracture rates in years 1-3 among pediatric and adult OI patients with off-label treatments. Rates decreased over the observation period with lower rates in the second and third years compared to each previous year.

Fracture trend from the first to third year was assessed among a subgroup of patients with at least three full 3 yr of complete data and treated with off-label treatment. Results were stratified by age groups (pediatric <18 yr and adults ≥18 yr). Fracture rates slowly decreased each year in both age groups (Figure 2). Fracture rates among OI pediatric patients decreased by 11% from year 1-2 (1.22-1.09) and by 20% from year 2-3 (1.09-0.87) (Figure 2). Among adult OI patients in the treated subgroup, fracture rate decreased by 16% from year 1 to year 2 (0.57-0.48) and by 10% from year 2 to year 3 (0.48-0.43).

Among all fracture episodes, tibia/fibula fractures were the most common (16.0%), followed by hip fractures (12.3%), forearm fractures (11.5%), femur fractures (10.6%), and vertebral fractures (9.4%) (Table 5). Tibia/fibula, femur, and hip fractures comprised almost half of all fracture episodes among OI patients age <7 yr; those aged 7-17 yr had highest occurrence in forearms (18%). Finger and toe fractures comprised 7% of all episodes and happened most among pediatric patients aged 7-17 yr (5.2% and 4.0%).

**Table 5:** Fracture episodes occurrence by body parts and age group among all OI patients.

	**Overall**		**<7**		**7-17**		**18-25**		**26-44**		**45-64**		**>65**	
**Site**	***N***	**%**	***N***	**%**	***N***	**%**	***N***	**%**	***N***	**%**	***N***	**%**	***N***	**%**
**Tibia_Fibula**	280	16.0	87	-	85	14.9	26	14.6	33	11.8	43	13.6	6	12.2
** Hip**	216	12.3	68	24.1	63	11.0	19	10.7	29	10.4	32	10.1	5	10.2
** Forearm**	202	11.5	30	18.8	104	18.2	18	10.1	25	9.0	19	6.0	6	12.2
** Femur**	186	10.6	59	8.3	60	10.5	16	9.0	25	9.0	22	7.0	4	8.2
** Vertebra**	165	9.4	12	16.3	48	8.4	17	9.6	33	11.8	49	15.5	6	12.2
** Foot**	132	7.5	21	3.3	43	7.5	14	7.9	29	10.4	23	7.3	2	4.1
** Humerus**	126	7.2	38	5.8	43	7.5	10	5.6	9	3.2	22	7.0	4	8.2
** Finger**	68	3.9	12	10.5	30	5.2	3	1.7	14	5.0	7	2.2	2	4.1
** Ribs**	62	3.5	7	3.3	7	1.2	10	5.6	14	5.0	22	7.0	2	4.1
**Hand_Wrist**	58	3.3	4	1.9	20	3.5	8	4.5	19	6.8	7	2.2	0	0.0
** Toe**	54	3.1	5	1.1	23	4.0	4	2.2	11	3.9	9	2.8	2	4.1
** Pelvis**	46	2.6	0	1.4	9	1.6	11	6.2	8	2.9	11	3.5	7	14.3
** Other**	37	2.1	7	0.0	3	0.5	4	2.2	6	2.2	17	5.4	0	0.0
** Clavicle**	31	1.8	5	1.9	12	2.1	5	2.8	3	1.1	6	1.9	0	0.0
**Knee_Patella**	27	1.5	1	1.4	5	0.9	3	1.7	7	2.5	9	2.8	2	4.1
** Scapula**	26	1.5	1	0.3	9	1.6	6	3.4	4	1.4	6	1.9	0	0.0
** Ankle**	17	1.0	0	0.3	2	0.3	0	0.0	9	3.2	6	1.9	0	0.0
** Head**	17	1.0	4	0.0	6	1.0	4	2.2	0	0.0	2	0.6	1	2.0
** Sternum**	5	0.3	0	1.1	0	0.0	0	0.0	1	0.4	4	1.3	0	0.0

### Musculoskeletal disorders

**Figure 3:**
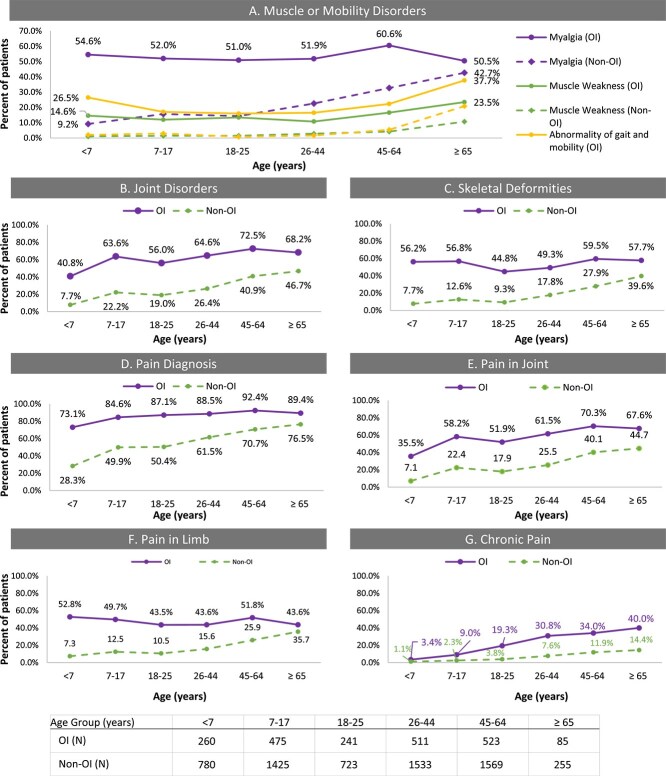
Frequency of musculoskeletal disorders by age groups in OI (solid lines) and non-OI (hashed lines) cohorts. Graphs display the proportion (%) of patients in each age group with muscle or mobility disorders (A), joint disorders (B), skeletal deformities (C), or pain (D-F). Except for chronic pain, these disorders were common among OI patients even in the youngest (<7 yr) age group and rare in the non-OI cohort in patients <45 yr. Chronic pain increased in frequency with age was more common among OI than non-OI in all age groups.

Muscle or mobility disorders (including myopathy, muscle atrophy, muscle weakness, myalgia, gout, abnormalities of gait, and mobility) were common among individuals with OI. Overall, 62.7% of patients with OI had mobility disorders, compared with 25.5% among the non-OI cohort. Myalgia was the most common muscle or mobility disorder among OI cohort (54.3% overall compared with 22.0% in non-OI cohort). More than half of patients with OI had myalgia at a young age (<7 yr), and the occurrence remained high across all age groups (Figure 3A). Myalgia occurred at a low frequency in the non-OI cohort, especially in younger age groups. Abnormality of gait and mobility occurred in 20.3% and 3.9% of the OI and non-OI cohorts, respectively. Gait abnormalities were most frequent in the OI cohort in the under 7 or ≥65 yr age groups and were rare in non-OI individuals until age ≥65 (Figure 3A). Muscle weakness occurred in 13.9% and 3.1% of the overall OI and non-OI cohorts, respectively.

Age group trends in musculoskeletal disorders were similar between the OI and non-OI cohorts, though OI patients had higher occurrence in all age groups (Figure 3A-C). Overall, 62.5% of the OI cohort and 26.7% of the non-OI cohort had joint disorders, while 54.2% and 17.8%, respectively, had skeletal deformities. The most common deformities among OI patients were: other acquired deformities of the limbs (9.1% OI vs 2.7% non-OI, highest in the OI <7 yr age group); deforming dorsopathies (22% OI vs 3% non-OI, highest in the OI 7-18 yr group); spondylosis (10% OI vs 4% non-OI, frequency increased with age in both OI and non-OI cohorts); and congenital malformation and deformation of the musculoskeletal system (16% OI vs 2% non-OI, highest in <7 yr age group).

Patients with OI had higher occurrence of diagnosed pain than the non-OI cohort, with the largest difference in the youngest (<7 yr) age group: 73% OI and 28% non-OI (Figure 3D). Pain in joints and pain in limbs were more common in the OI group, even in the youngest age group (Figure 3E and F). The occurrence of pain in joint and chronic pain increased with age (Figure 3E and G). Chest pain, abdominal/pelvic pain, dorsalgia, and acute pain were also common among patients with OI, and the overall occurrences were 23.3%, 32.0%, 47.0%, and 11.7%, respectively (data not shown). Diagnosed pain and occurrences were also more common among the OI-treated cohort than the non-OI cohort but showed no consistent pattern of being more or less frequent than the overall OI cohort across age groups (Table S2).

**Figure 4:**
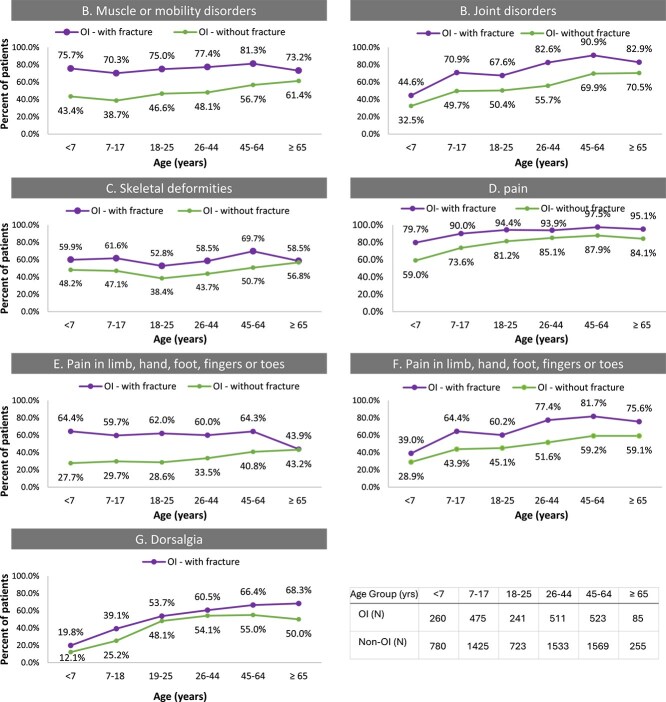
Frequency of musculoskeletal disorders by age groups among OI patients with or without fracture. Graphs display the proportion (%) of patients in each age group with muscle or mobility disorders (A), joint disorders (B), skeletal deformities (C), or pain (D-G). There was a high occurrence of musculoskeletal disorders in OI patients regardless of fracture status. Pain diagnosis was the most common disorder reported for both groups.

Although more frequent among OI patients who experienced fracture, musculoskeletal disorders were common even among OI patients without fracture (Figure 4). Osteogenesis imperfecta patients without fracture experienced muscle mobility disorders, joint disorders, and skeletal deformities even among the youngest age group (Figure 4A-C). Pain was also diagnosed frequently in OI patients regardless of fracture status (Figure 4D). Dorsalgia and joint pain appeared less associated with fracture status, while pain in long bones was substantially more common among those with fracture, especially in the younger age groups (Figure 4E and F).

## Discussion

This retrospective US cohort analysis provides data on fractures and other musculoskeletal disorders for 2095 OI patients, 35% of whom were pediatric patients. About one quarter of the overall OI cohort had treatment with bisphosphonates, denosumab, or teriparatide. Romosozumab was assessed but no prescriptions were found among this cohort of patients. The majority of the treatment was IV bisphosphonates, followed by oral bisphosphonates. Prescriptions of denosumab and teriparatide were scarce in patients under age 45. A subsequent study in this same cohort assessed prescriptions for pain and psychiatric medications and healthcare resource utilization; these results are published separately.^25^

Fracture rates during the study period were higher among OI treated patients than the overall OI cohort (28% vs 13%). The treatment rate in this study was consistent with a recent US claims data study that combined another commercial database with state Medicaid and Medicare data, which reported treatment with bisphosphonates, denosumab, romosozumab, and PTH analogs in 23% of all OI patients.^26^

More than half of pediatric patients, 35% of young adult (18-25 yr) and 31% of older adult patients (>25 yr) with OI experienced at least one fracture within a year. Among those with ≥1 fracture, 33% of pediatric patients, 27% of young adults and 21% of older adults had ≥3 fractures in a year, indicating a more severe status of their disease. Fracture occurrence among the treated OI subgroup was similar to the overall OI cohort, with more pediatric and young adult patients experiencing 3 or more fractures in a year (42% and 40% among patients with fractures). Osteogenesis imperfecta patients had much higher fracture occurrence and annual fracture rate compared with those in the non-OI population (40% vs 3%; 0.84 vs 0.03 in the first year of analysis). Fracture patterns by patient age were as expected for OI, with the highest fracture rate among pediatric patients, especially those under age 7 yr (1.4 per patient per year), and the lowest fracture rate in those 26-44 yr, followed by a slight increase among those 45 yr and older.

The overall fracture rate in this study (average 0.84 first year, 0.58 during entire CE) was slightly lower than the fracture rate reported in a previous US study conducted through the Brittle Bone Disorder Consortium (BBDC), which showed an average fracture rate of 1.03 (SD 2.89) across all OI subtypes.^24^ However, BBDC data were largely skewed to a younger OI population (median age 12.6 vs 28 in our study) with a higher frequency of off-label treatment with bisphosphonates.

Fracture rates reported here were substantially higher than fracture rates reported from a Danish nation-wide registry study.^11^ We observed annualized fracture rates of 1.04, 0.82, and 0.50 among OI patients aged <7, 7-17, and 18-26 yr, respectively, compared to 0.23 fractures per person-year for ages 0-19 in the Danish cohort. Several factors may explain this difference. (1) Fracture identification methods differed—our study used ICD-coded diagnoses from reimbursement data, while the Danish study relied on patient interviews, which are subject to recall bias. (2) Fracture counting criteria also varied—our study used a 90-d rolling window per body part (grouping same-site fractures within 90 d as a single event), while the Danish study applied a 180-d washout for the same diagnosis. (3) Differences in follow-up duration likely contributed—our study had a shorter follow-up with a higher early fracture rate, potentially inflating the overall rate compared to the longer follow-up in the Danish cohort. (4) Cultural and healthcare system differences may also influence fracture reporting and care-seeking behavior (eg, hospital vs home treatment). Despite these differences, both studies showed a consistent age-related pattern, with the highest fracture burden in children, a decline through young adulthood, and a rise again after age 45-50 yr.

The median fracture rates during the entire CE for the pediatric age groups (<7 yr and 7-17 yr) were 0.59 and 0.44 for all OI patients, and 1.09 ad 0.49 for OI patients with off-label treatments; adult median is 0. This result is in line with the baseline fracture rate of 0.7 per year reported in a recent clinical trial for OI.^27^ Considering the population difference, our fracture rate findings were consistent with previous reports. Among patients with ≥1 fracture, the median number of fractures per year ranges from 2 for pediatric and elderly patients (65+ years) to 1 for other adults.

The subgroup of OI patients with off-label treatment had an average fracture rate of 0.98 within 1 yr of treatment and had higher fracture rates across all age groups, especially for those under 7 and those aged 26-44 yr. However, this subgroup of patients was not matched with a no-treatment group and might be biased toward more severe types of OI. Among the subgroup of OI patients with off-label treatment and with full 3 yr of available data, fracture rates slowly went down from the first year to the third year (annual reductions ranged from 10% to 20%). This trend was consistent across both pediatric and adult patients. However, even 3 yr post off-label treatment, OI patients continued to experience high fracture rates, underscoring the unmet need in current disease management. These results should be interpreted with caution as the patient selection methods required at least two OI codes for indexing, with the first diagnosis code serving as the index date. As a result, all patients had at least one OI code in the first follow-up year. It is possible that some OI diagnoses were recorded alongside fracture events, potentially inflating the fracture rate in Year 1. While years 2 and 3 may offer a more stable fracture rate estimate due to the study design, these rates could still be underestimated due to reliance on diagnostic and billing codes. Studies have shown that patients with OI experience the highest fracture rate in early life, which decreases with age.^11^^,^^28^ The trend of decreasing fracture rates observed in this analysis may therefore reflect the natural progression of the disease. Additionally, the year 1 to year 3 trend analysis was limited to patients with three full years of CE, potentially biasing the results toward a subgroup of more stabilized or milder cases who did not require Medicaid/Medicare transitions. Interpretation of this subgroup analysis should be approached with caution due to potential selection bias and the smaller sample size. As a result, the findings may not be generalizable to the broader OI population with off-label treatment or the general OI population.

Vertebral fractures were common for OI patients comprising 9% of all fractures but were less frequent among the <7 yr group (3%). This occurrence rate is lower than the vertebra fracture rates observed in clinical study settings, possibly because vertebral fractures can occur without significant pain or other noticeable symptoms (morphometric vertebral fractures) and can be difficult to identify with standard imaging.^29^ Osteogenesis imperfecta patients had much higher rates of muscle/mobility disorders and skeletal deformities starting at young age compared with the non-OI cohort. More than half of the patients, including younger pediatric patients, had diagnosed myalgia or skeletal deformities, while occurrence among general population was low (<30%) until age 45 and above.

Diagnosed pain was very common among OI patients of all age groups (86.5% overall). The results were consistent with a recent patient survey study, where more than 80% of adult OI patients reported pain within the previous 12 mo.^9^ This is also in line with a previous cross-sectional study where musculoskeletal pain was present among 86% of the patients.^30^ Some of the off-label OI-therapies are thought to improve pain outcomes and may have been prescribed for pain as insurance claims do not differentiate the reason for treatment. Rates of pain diagnosis and specific types of pain among the OI-treated cohort were also high and varied in comparison to the overall OI cohort across measures and age groups.

Even among the youngest patients with the lowest pain diagnosis rate (those <7 yr), the occurrence of diagnosed pain was at 73% (vs 28% of general population). The most common types of pain across all age groups were pain in limbs and pain in joints, each reported in more than half of pediatric patients and continuing or increasing occurrence with age. Dorsalgia and chronic pain were less common among young pediatric OI patients but increased with age and were much higher than the general population. This is consistent with a multicenter study evaluating pain in OI, though chronic pain was reported at a higher frequency in the multicenter study indicating the population evaluated might be more severe than the population in our analysis.^7^

Patients with fractures had a higher rate of musculoskeletal disorders at all ages compared with those without fractures. The most notable difference was for pain in limbs, which occurred in about 60% of patients with fracture and 30%-40% of those without fracture. The results show that pain is frequent in OI even without fracture, thus, while managing/preventing fractures could largely reduce pain for patients with OI, better pain management is also needed outside of the pain reduction that may be achieved by agents reducing fracture. Osteogenesis imperfecta patients had a high frequency of musculoskeletal disorders regardless of fracture occurrence. Addressing those conditions in addition to fracture prevention is required to offer a full management strategy for all symptoms in OI patients.^4^

### Limitations

The study and conclusions are limited by the predominance of private insurance among the study population, the retrospective data collection, and the lack of distinction between different types of OI. The data-source does not represent well the Medicaid and Medicare population, which may include more disabled or more severely affected patients. Therefore, fracture rates/occurrences and disorders might be lower among patients in this database than in the general OI population. Due to lack of Medicare data and small sample size among individuals ≥65 yr, the results in this age group cannot be generalized to the US population. Despite the sample bias limitation, this data is well-representative of the commercially insured population in the US; treatment pattern is also consistent with other US studies with broad commercial, Medicaid and Medicare populations.

The long-term fracture burden of OI could not be estimated in this study due to the limited study period and mostly commercial patient population. In claims data, payment records are based on diagnostic coding that may be driven by reimbursement concerns and may or may not accurately reflect the true medical condition. Accuracy of diagnosis also depends on accuracy of ICD-10 coding and are subject to under-reporting and misreporting. Osteogenesis imperfecta subtype and severity information is not available in the results since OI patients were only identified with a single ICD-10 coding. However, musculoskeletal disorders were assessed by fracture status, which was used as a proxy of severity. Fracture occurrences identified within a 90-d period were assessed as the same event. In practice, the same site may re-fracture in this time frame and lead to undercounting of fractures in this study; or the same fracture might be treated for years, possibly leading to overcounting of fractures. Fractures that were not diagnosed using ICD-10 codes were not included in this study (eg, home managed fractures, which is common for OI patients, asymptomatic fractures, or fractures that did not require treatments or procedures). Thus, the total fracture burden of OI patients is likely to be underestimated in our analyses. Likewise, musculoskeletal disorders may also be under-reported in ICD-10 codes. However, the diagnosis of OI might not be systematic or comprehensive in claims data and might be coded when there is a treatment need (eg, fracture) or a routine checkup of the disease. This could potentially result in a selection bias of more severe OI patients in this database thus overestimating fracture burden of OI overall. This could be especially true for adult OI population who have less frequent routine checkups and lower risk of fracture.

Results among the OI cohorts grouped by treatment status do not provide information on the cause of any differences observed between groups. No conclusions about the effectiveness of off-label therapies on pain or fracture occurrences can be made from these analyses as the groups were not matched for status, subtypes or severity, and the data collection methods were not designed for such analyses.

### Conclusions

Patients with OI had a high occurrence of fractures and high fracture rates, especially among pediatric patients. Limb, hip, and vertebrae fractures were most common among patients with OI. Osteogenesis imperfecta patients treated with off-label medications, and particularly the pediatric age groups, were still experiencing a high number of fractures post treatment, highlighting the need for better fracture management.

Osteogenesis imperfecta patients experience high occurrence of musculoskeletal disorders across all ages and starting at much younger ages than the first appearance of these disorders in the non-OI cohort. Diagnosed pain was prevalent among most OI patients with pain in limbs and joints the most dominant types of pain. Patients with fractures had higher rates of musculoskeletal disorders, but rates were still high even in patients without fracture. Fracture prevention might decrease pain in limbs and joints for OI patients.

## Supplementary Material

FigureS1_Yangetal_20Aug2025_S1_ziaf124

FigureS2_Yangetal_20Aug2025_S2_ziaf124

Supplementary_Materials_Yangetal_OIFracture_Tables_S1and_S2

## Data Availability

This was a claims database analysis using IQVIA PharMetrics Plus closed claims data obtained under license from IQVIA Inc. The raw data cannot be publicly shared since it was obtained from IQVIA and as per signed agreement between Ultragenyx and IQVIA Inc. However, we have provided all relevant data in the manuscript that supports the research objectives and conclusions. We confirm that interested researchers can reach out to IQVIA Inc. to access the data.
