# Supplementary material for: Real-world data of fracture rates and musculoskeletal disorders for patients living with osteogenesis imperfecta
Source: JBMR Plus. 2025 Jul 21;9(10):ziaf124. doi: 10.1093/jbmrpl/ziaf124 (PMC12445870; doi:10.1093/jbmrpl/ziaf124)
Supplement: FigureS1_Yangetal_20Aug2025_S1_ziaf124 [file figures1_yangetal_20aug2025_s1_ziaf124.pdf]

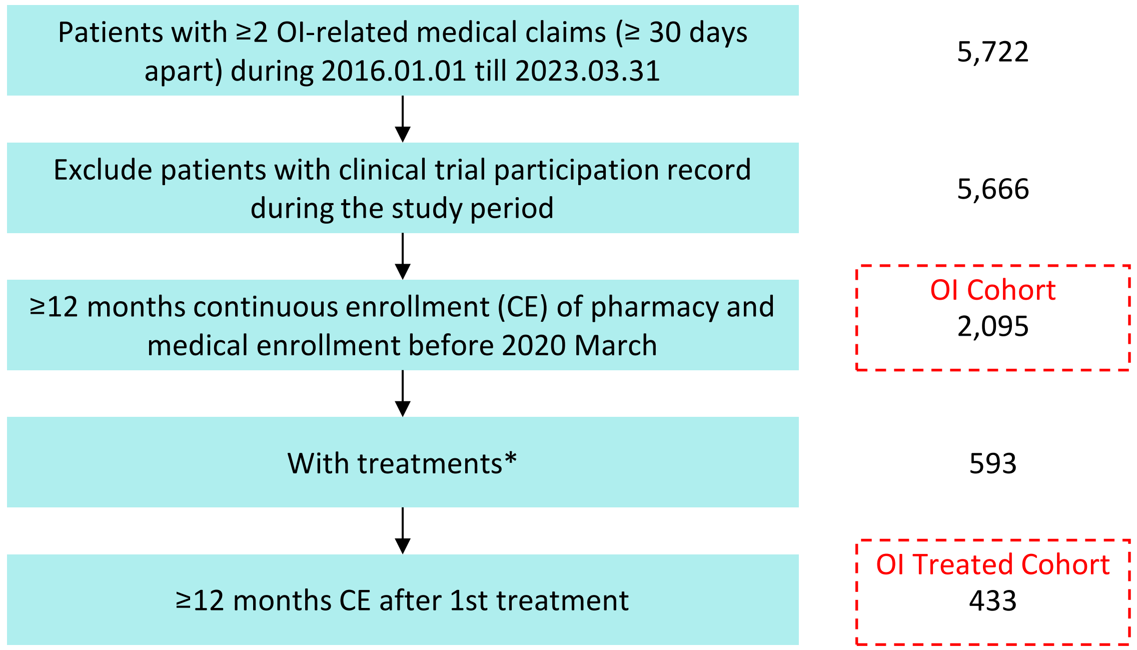

Figure S1. OI patient selection. Flow chart outlines the selection process for the OI study cohort of 2,095 OI patients and the OI-treated subgroup.
