# Supplementary material for: Real-world data of fracture rates and musculoskeletal disorders for patients living with osteogenesis imperfecta
Source: JBMR Plus. 2025 Jul 21;9(10):ziaf124. doi: 10.1093/jbmrpl/ziaf124 (PMC12445870; doi:10.1093/jbmrpl/ziaf124)
Supplement: FigureS2_Yangetal_20Aug2025_S2_ziaf124 [file figures2_yangetal_20aug2025_s2_ziaf124.pdf]

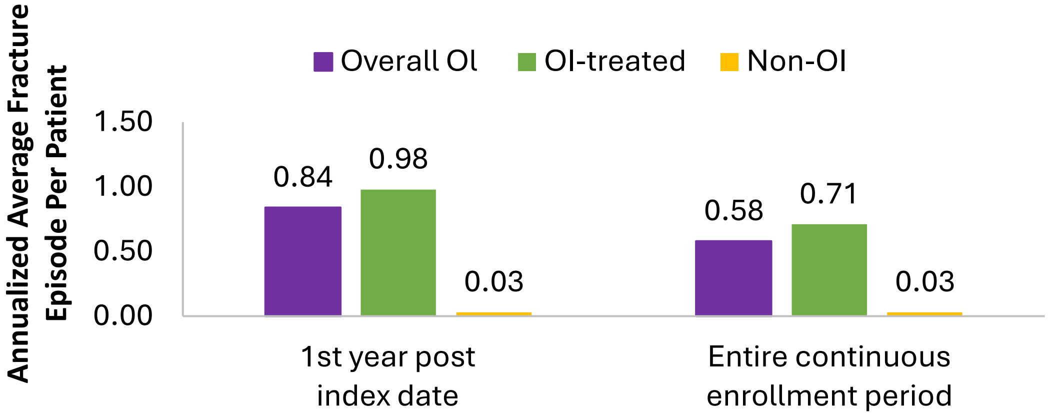

	During 1st year				During entire CE		
	All OI (n=2095)	OI-treated (n=433)	Non-OI (n=6285)		All OI (n=2095)	OI-treated (n=433)	Non-OI (n=6285)
N patients w/ fx	842	194	164		1082	268	474

Figure S2. Annualized number of fractures, 1st year post index and entire continuous enrollment period. Graph displays the annualized average number of fractures in the Overall OI, OI-treated, and non-OI cohorts over the first-year post index date and the entire CE period. Fractures were common among overall OI patients over both periods (>0.5 per patient annually), and more common for the OI-treated subgroup (>0.71). Fractures were very rare (0.03 per patient annually over both time periods) for the non-OI cohort. The table provides the corresponding total number of fracture episodes for each patient group and time period.
