# Supplementary material for: Real-world data of fracture rates and musculoskeletal disorders for patients living with osteogenesis imperfecta
Source: JBMR Plus. 2025 Jul 21;9(10):ziaf124. doi: 10.1093/jbmrpl/ziaf124 (PMC12445870; doi:10.1093/jbmrpl/ziaf124)
Supplement: Supplementary_Materials_Yangetal_OIFracture_Tables_S1and_S2 [file supplementary_materials_yangetal_oifracture_tables_s1and_s2.docx]

**Table S1. Demographics**

|  | **OI Patients (N = 2095)** | | **Matched non-OI cohort (N = 6285)** | | **Treated OI Patients (N=433)** | |
| --- | --- | --- | --- | --- | --- | --- |
| **Demographics** | **Mean/N/Median [SD/%/IQR]** | **SD/%/IQR** | **Mean/N/Median** | **SD/%/IQR** | **Mean/N/Median** | **SD/%/IQR** |
| **Age (years)** |  |  |  |  |  |  |
| Age (mean, SD) | 30.50 | 20.20 | 30.63 | 20.60 | 31.63 | 22.71 |
| Age (median, IQR) | 28 | 13, 48 | 28 | 13, 48 | 26 | 11, 54 |
| **Gender (n, %)** |  |  |  |  |  |  |
| Male | 929 | 44.3% | 2787 | 44.3% | 193 | 44.6% |
| Female | 1166 | 55.7% | 3498 | 55.7% | 240 | 55.4% |
| Sex Unknown | 0 | 0.0% | 0 | 0.0% | 0 | 0.0% |
| **Region (n, %)** |  |  |  |  |  |  |
| Northeast | 352 | 16.8% | 1065 | 16.9% | 61 | 14.1% |
| Midwest | 549 | 26.2% | 1595 | 25.4% | 129 | 29.8% |
| South | 834 | 39.8% | 2415 | 38.4% | 176 | 40.6% |
| West | 347 | 16.6% | 1151 | 18.3% | 66 | 15.2% |
| Region Unknown | 13 | 0.6% | 59 | 0.9% | 1 | 0.2% |
| **Insurance** |  |  |  |  |  |  |
| Medicare | 78 | 3.7% | 234 | 3.7% | 20 | 4.6% |
| Commercial | 1825 | 87.1% | 5475 | 87.1% | 394 | 91.0% |
| Medicaid | 187 | 8.9% | 561 | 8.9% | 19 | 4.4% |
| Other | 5 | 0.2% | 15 | 0.2% | 0 | 0.0% |
| **Enrollment duration (years)** |  |  |  |  |  |  |
| Years (mean, SD) | 3.40 | 2.03 | 3.17 | 2.09 | 3.97 | 1.97 |
| Years (median, IQR) | 2.91 | 1.51, 5.16 | 2.42 | 1.33, 4.75 | 3.92 | 2.01, 5.61 |
| **Year of Index Date (n, %)** |  |  |  |  |  |  |
| 2016 | 1140 | 54.4% | 4249 | 67.6% | 261 | 60.3% |
| 2017 | 499 | 23.8% | 859 | 13.7% | 101 | 23.3% |
| 2018 | 370 | 17.7% | 842 | 13.4% | 61 | 14.1% |
| 2019 | 86 | 4.1% | 335 | 5.3% | 10 | 2.3% |

**Table S2 Pain and Musculoskeletal Complications in treated OI patients**

| **Pain or Complication** | **All (N=433)** | **<7 years (N=63)** | **1-18 years (N=124)** | **19-25 years (N=28)** | **26-44 years (N=47)** | **45-64 years (N=146)** | **≥65 years (N=25)** |
| --- | --- | --- | --- | --- | --- | --- | --- |
| **Muscle or mobility disorder** | 244 (56.4%) | 42 (66.7%) | 70 (56.6%) | 16 (57.1%) | 23 (48.9%) | 77 (52.7%) | 16 (64.0%) |
| Myopaty | 4 (0.9%) | 1 (1.6%) | 2 (1.6%) | 1 (3.6%) | 0 | 0 | 0 |
| Muscle atrophy | 6 (1.4%) | 1 (1.6%) | 1 (0.8%) | 1 (3.6%) | 0 | 3 (2.1%) | 0 |
| Muscle Weakness | 56 (12.9%) | 11 (17.5%) | 18 (14.5%) | 5 (17.9%) | 4 (8.5%) | 15 10.3%) | 3 (12.0%) |
| Myalgia | 198 (45.7%) | 34 (54.0%) | 56 (45.2%) | 13 (46.4%) | 20 (42.6%) | 64 (43.8%) | 11 (44.0%) |
| Gout | 1 (0.2%) | 0 | 0 | 0 | 0 | 1 (0.7%) | 0 |
| Abnormalities of gait and mobility | 73 (16.9%) | 12 (19.1%) | 20 (16.1%) | 5 (17.9%) | 9 (19.2%) | 18 (12.3%) | 9 (36.0%) |
| **Joint disorders** | 245 (56.6%) | 25 (39.7%) | 66 (53%) | 13 (46.4%) | 31 (66.0%) | 94 (64.4%) | 16 (64.0%) |
| **Skeletal deformities** | 235 (54.3%) | 41 (65.1%) | 85 (68.6%) | 16 (57.1%) | 21 (44.7%) | 61 (41.8%) | 11 (44.0%) |
| **Pain Dx*** | 352 (81.3%) | 48 (76.2%) | 96 (77.4%) | 20 (71.4%) | 44 (93.6%) | 123 (84.3%) | 21 (84.0%) |
| Pain in limb | 184 (42.5%) | 33 (52.4%) | 53 (42.7%) | 13 (46.4%) | 18 (38.3%) | 57 (39.0%) | 10 (40.0%) |
| Pain in joint | 200 (46.2%) | 21 (33.3%) | 55 (44.4%) | 12 (42.9%) | 25 (53.2%) | 74 (50.7%) | 13 (52.0%) |
| Dorsalgia | 149 (34.4%) | 10 (15.9%) | 31 (25.0%) | 7 (25.0%) | 23 (48.9%) | 64 (43.8%) | 14 (56.0%) |
| Chronic pain | 83 (19.2%) | 4 (6.4%) | 11 (8.9%) | 6 (21.4%) | 17 (36.2%) | 39 (26.7%) | 6 (24.0%) |
| *Not all pain subtypes are listed. Overall pain diagnosis also included chest pain, acute pain, pelvic and perineal pain, abdominal pain, headache, and pain not specified. | | | | | | | |
